# A Case of Delayed Paraplegia Following Missed Diagnosis on Computed Tomography

**DOI:** 10.7759/cureus.4151

**Published:** 2019-02-28

**Authors:** William Clifton, Gazanfar Rahmathulla

**Affiliations:** 1 Neurosurgery, Mayo Clinic, Jacksonville, USA; 2 Neurosurgery, University of Florida College of Medicine, Jacksonville, USA

**Keywords:** trauma, fracture, tlics, spine, plc, vertebral body

## Abstract

There are many proposed classification systems for traumatic thoracolumbar fractures (TLF). More recently published are the AO Spine Classification System and the Thoraco-Lumbar Injury Classification System (TLICS). There has been a paucity of high-level evidence to link these classification system subtypes with clinical outcomes and/or management strategies. Previously, post-traumatic burst fractures or two column injuries identified on computed tomography (CT) scan have been deemed stable injuries. The addition of magnetic resonance imaging (MRI) evaluation for concomitant ligamentous injuries in cases of incomplete burst fractures has been widely debated without high-level evidence. In this report, we present a case of an incomplete burst fracture at L1, AO-A3, which did not receive an MRI and presented with delayed paraplegia four weeks later.

## Introduction

Several classification systems have been proposed for traumatic thoracolumbar fractures (TLF). The more recently published systems include the AO Spine Classification System and the Thoraco-Lumbar Injury Classification System (TLICS) [1-4]. There has been a paucity of high-level evidence to associate these classification system subtypes with imaging findings, clinical outcomes, management strategies, and/or surgical approaches. Previously, post-traumatic burst fractures or two column injuries identified on computed tomography (CT) scan have been deemed stable injuries, not necessitating urgent surgical intervention [5-7]. The addition of MRI evaluation for concomitant ligamentous injuries in cases of incomplete burst fractures has been widely debated without high-level evidence [8-12]. In this report, we present a case of a reported two-column (anterior and middle) fracture at L1 diagnosed by CT; however, the patient did not receive an initial magnetic resonance imaging (MRI) and subsequently presented with delayed paraplegia four weeks later. MRI revealed extensive ligamentous injury and subluxation at L1-L2 along with compression of his conus. This case highlights the importance of establishing a consensus on the radiographic assessment of thoracolumbar fracture morphology, as well as what fracture types should receive MRI evaluation for ligamentous injury.

## Case presentation

**Figure 1:**
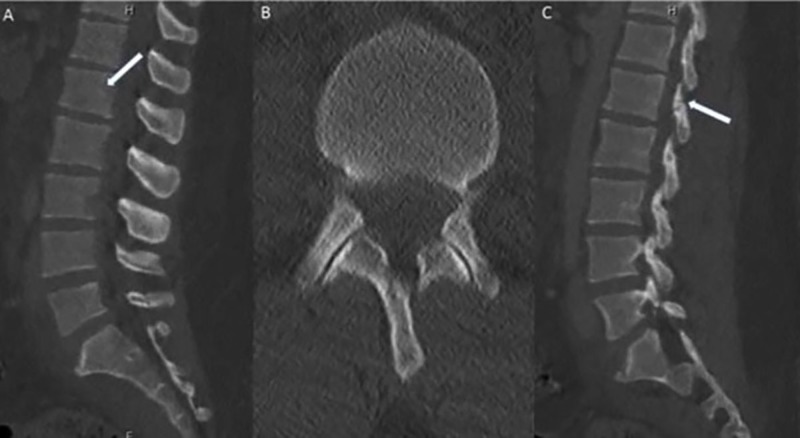
Admission CT scan (A) There is normal vertebral body alignment with a subtle fracture through the superior endplate of L1 extending to the posterior aspect of the vertebral body (white arrow). (B) Axial image of the L1-2 facet joints that show no sign of widening or abnormality. (C) Sagittal image showing linear hypodensity within the pars interarticularis of L1 concerning for acute fracture that was not seen on other reconstructions (white arrow). This was not reported on the original CT read. CT: computed tomography

**Figure 2:**
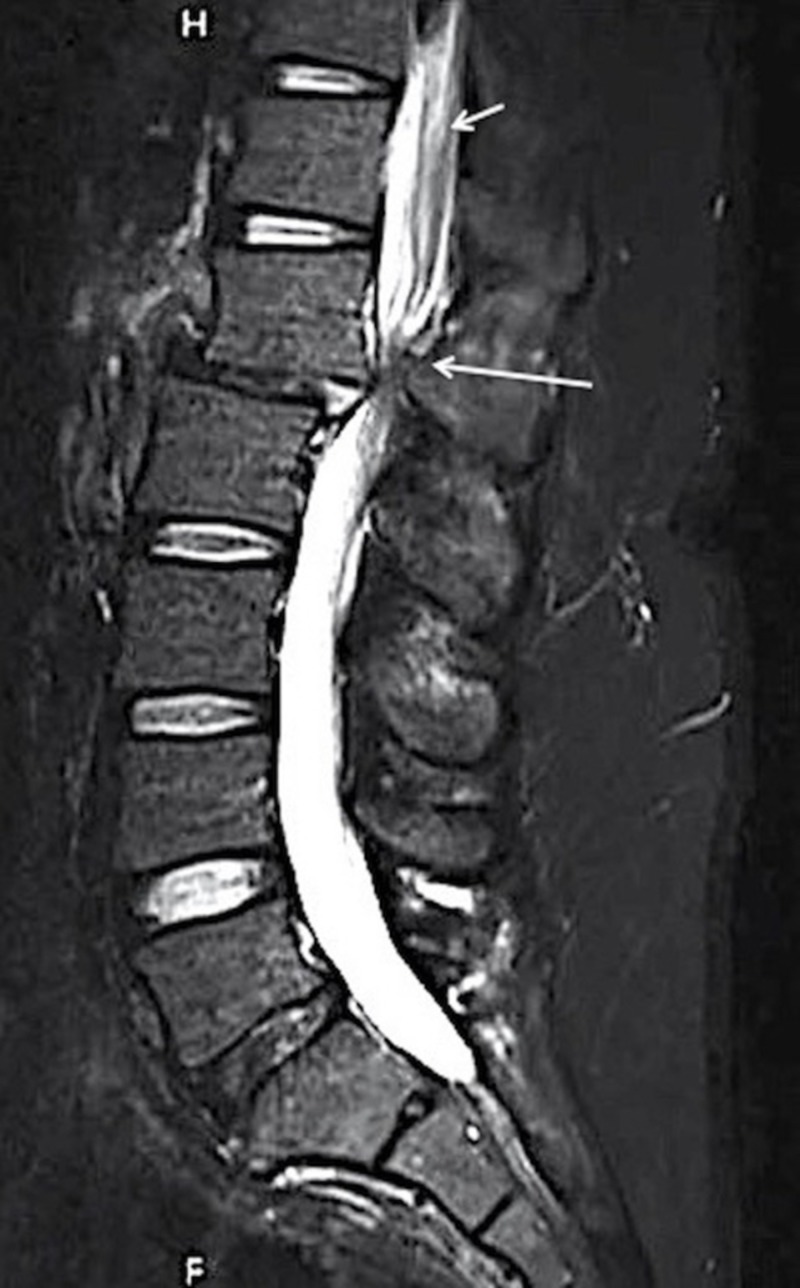
MRI L spine post mobilization L1-2 distraction injury and severe canal compromise from ligamentous damage (long white arrow). Extensive conus and cord T2 hyperintensity is shown (short white arrow), suggesting vascular injury from severe venous compression at the L1-2 level. MRI: magnetic resonance imaging

**Figure 3:**
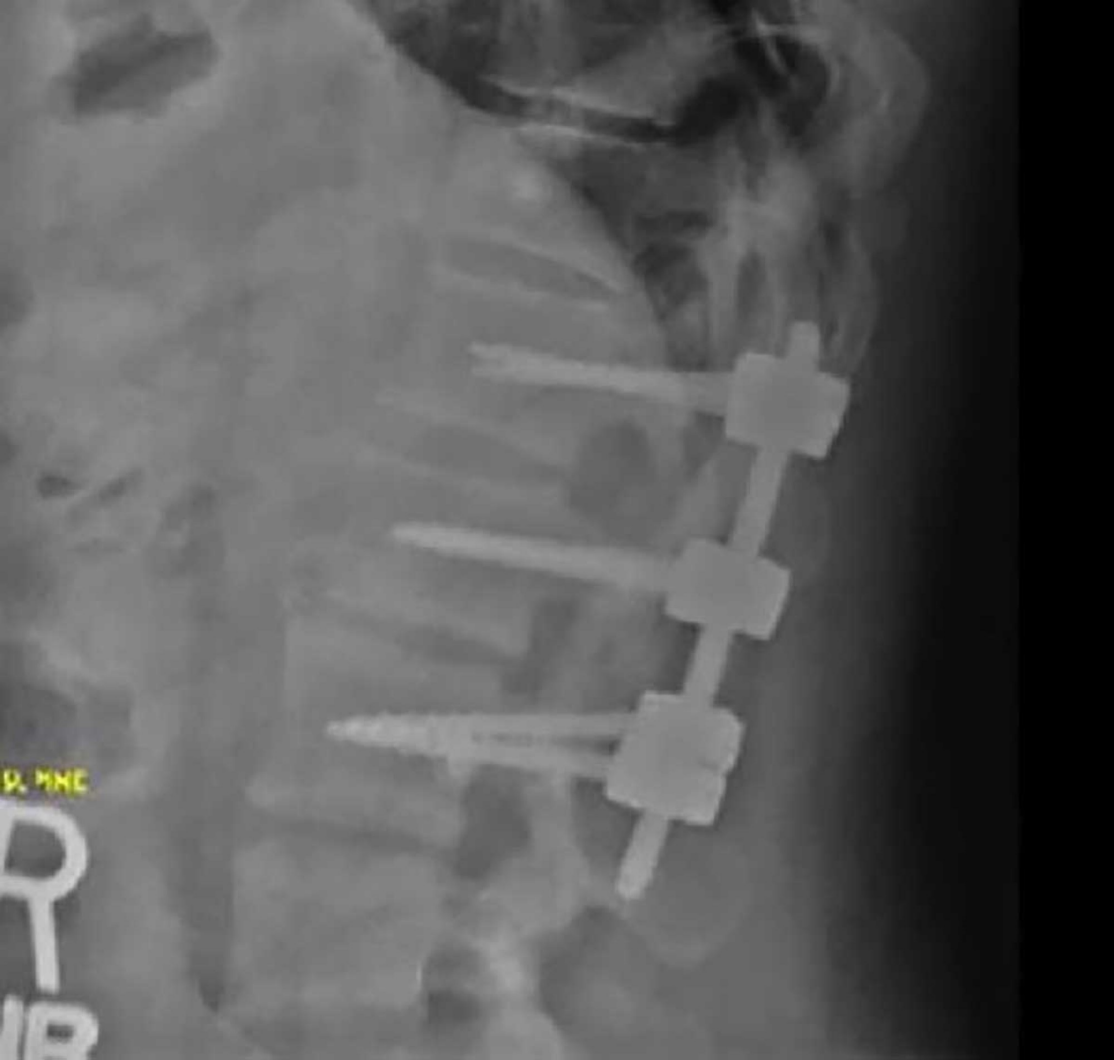
Postoperative lateral X-ray Good hardware placement and satisfactory reduction of the previously seen L1-2 distraction injury is seen.

A 28-year-old male was admitted after a motor vehicle collision (MVC) with low back pain and orthopedic fractures. The admission CT scan of his lumbar spine was read as a posterior superior endplate fracture at L1 extending to the posterior vertebral body, without posterior element displacement or disc space widening (Figure 1). The patient was placed in a thoracolumbosacral orthosis (TLSO). MRI was deferred at the time due to an emergent orthopedic procedure for bilateral open fractures of the lower extremities. The patient remained in the hospital for four weeks with immobilization due to his orthopedic procedures. He was not able to obtain an MRI during this period due to the external orthopedic fixation. He did not complain of any neurologic symptoms, was voiding independently and able to wiggle his toes in the orthopedic fixation. When he was released from fixation and finally mobilized the patient had sudden and severe leg weakness both proximally and distally accompanied by paresthesias. An MRI (Figure 2) showed complete ligamentous disruption through the disc space and posterior ligamentous complex (PLC) disruption with subluxation of the vertebral bodies, AO L1/2 type C2, L1 type A3. There was significant edema in the conus that extended up into the thoracic spinal cord concerning for ischemic injury secondary to severe compression. The patient underwent emergent open decompression at L1-2 and pedicle screw fixation at T12-L2 (Figure 3). The displaced segment was carefully reduced under fluoroscopic guidance using rod distraction. The patient did not recover the motor function of his legs two months later at his last follow-up. His sensory symptoms improved and he had preserved genitourinary function.

## Discussion

Burst fractures compromise the most commonly encountered TLF [13-14]. The Denis model classifies these fractures as two column injuries on CT, which have been classically deemed as “stable” injuries. CT has been shown to have a high sensitivity and specificity for TLF [15]. CT findings are generally used to lead practitioners to order advanced imaging such as MRI to determine the presence of ligamentous injury. The timing of MRI acquisition after a fracture has been identified on CT has not been clearly defined by evidence-based studies. The AO spine classification system provides a thorough analysis of fracture morphology on CT and allows for prediction of ligamentous injury that can be confirmed with subsequent MRI. Incomplete burst fractures with ligamentous injury become dangerous for neural element impingement after the patient has been mobilized [16]. Fractures that involve the posterior edge of the vertebral body may also have significant stability compromise once the patient is load bearing. The early use of MRI may benefit patients with these injuries; however, specific evidence-based guidelines have not yet been developed. In the case of our patient, his CT on presentation was initially read as an incomplete burst fracture involving the superior endplate; however, there were subtle signs of facet and posterior element fracture, which were not obvious on the initial CT and only discovered upon retrospective imaging review. After several weeks of bedrest for leg traction, the patient had subluxation due to extensive anterior and posterior ligamentous complex injury. Although the patient had not yet been fully mobilized, the unrecognized ligamentous injury was severe enough to cause subluxation from the minimal movement of the patient’s spine. If the MRI had been acquired at the time of presentation, the ligamentous injury would have been identified and the patient would have undergone spinal fixation prior to his orthopedic procedures. Although this singular case does not provide sufficient evidence to strongly suggest a universal standard for advanced neuroimaging in the setting of all thoracolumbar trauma, it provides an example of the potential for missed diagnoses of posterior element compromise in patients with burst fractures diagnosed on CT. Concomitant severe ligamentous injuries may present insidiously without obvious findings on this imaging modality. The biomechanics of suspected AO-A3 and AO-A4 type fractures provide reasonable suspicion for possible ligamentous complex injury as they involve the posterior portion of the vertebral body, indicating the potential for posterior ligamentous complex involvement and thus immediate or delayed neurologic compromise [17-20].

## Conclusions

TLF are common injuries that occur after trauma. There are several classification systems that attempt to provide guidance for diagnostic and clinical management; however, there is little high-level evidence supporting these classification systems and their significance. In this case, a seemingly stable posterior vertebral body injury seen on CT was discovered to have significant ligamentous injury and instability after the MRI was obtained post mobilization. CT imaging alone may not be a completely reliable imaging modality to fully assess spinal integrity in cases of posterior vertebral body fractures. Patients with coinciding ligamentous injuries may present radiographically with only subtle findings on CT that can be easily missed with catastrophic neurologic consequences. Further outcome-based studies using these classification systems and imaging modalities should be performed in order to understand their significance in the management of patients with acute traumatic thoracolumbar fractures.
